# Family doctors’ roles and perceptions on antibiotic consumption and antibiotic resistance in Romania: a qualitative study

**DOI:** 10.1186/s12875-023-02047-z

**Published:** 2023-04-10

**Authors:** Ioana Ghiga, Emma Pitchforth, Cecilia Stålsby Lundborg, Anna Machowska

**Affiliations:** 1 Department of Global Public Health, Karolinska Institutet, 171 77 Stockholm, Sweden; 2 Primary Care Research Group, University of Exeter Medical School, St Luke’s Campus, Heavitree Road, Exeter, EX1 2LU UK

**Keywords:** Antibiotic resistance, Antimicrobial resistance, General practitioners, Family doctors, Perceptions, Roles, Romania

## Abstract

**Background:**

Antimicrobial resistance (AMR) is a major global health issue, bringing significant health burden and costs to societies. Increased antibiotic consumption (ABC) is linked to AMR emergence. Some of the known drivers of ABC are antibiotics over-prescription by physicians and their misuse by patients. Family doctors are recognised as important stakeholders in the control of ABC as they prescribe antibiotics and are considered a reliable source of medical information by patients. Therefore, it is important to explore their perceptions, especially in Romania, which has the highest ABC among European Union Member States. Furthermore, there is no published research exploring Romanian family doctors’ perceptions regarding this phenomenon.

**Methods:**

This was a qualitative study with data collection via semi-structured interviews among 12 family doctors. Manifest and latent content analysis was used to gain an in-depth understanding of their perceptions. Findings were mapped onto the domains of the Behaviour Change Wheel to facilitate a theory driven systematization and analysis.

**Results:**

Two main subthemes emerged: i) factors affecting ABC and prescribing and ii) potential interventions to tackle ABC and antibiotic resistance. The factors were further grouped in those that related to the perceived behaviour of family doctors or patients as well as those that had to do with the various systems, local contexts and the COVID-19 pandemic. An overarching theme: ‘family doctors in Romania see their role differently when it comes to antibiotic resistance and perceive the lack of patient education or awareness as one of the major drivers of ABC’ was articulated. The main findings suggested that the perceived factors span across the capability, opportunity and motivational domains of the behaviour change wheel and could be addressed through a variety of interventions – some identified by the participants. Findings can also be viewed through cultural lenses which shed further light on the family doctor- patient dynamic when it comes to antibiotics use.

**Conclusion:**

Potential interventions to tackle identified factors emerged, revolving mostly on efforts to educate patients or the public. This exploratory research provides key perspectives and facilitates further research on potential interventions to successfully address AMR in Romania or similar settings.

**Supplementary Information:**

The online version contains supplementary material available at 10.1186/s12875-023-02047-z.

## Background

Antimicrobial resistance (AMR) is a major issue globally, bringing significant health burden and high costs to societies. In the European Union (EU), AMR is estimated to lead to 33,000 deaths per year and €1.5 billion yearly losses due to healthcare and productivity costs [1]. While several policy instruments were developed to control this phenomenon [2–4]data across EU Member States, show wide variation in antibiotic consumption and resistance rates [5–7].

Romania, who joined the EU in 2007, is among the top most affected countries with over 3% of its population using antibiotics daily [8]. Based on latest data from 2021, Romania ranks first among all EU members states when it comes to consumption of antibacterials for systemic use in community and hospital sector [7].

**Fig. 1:**
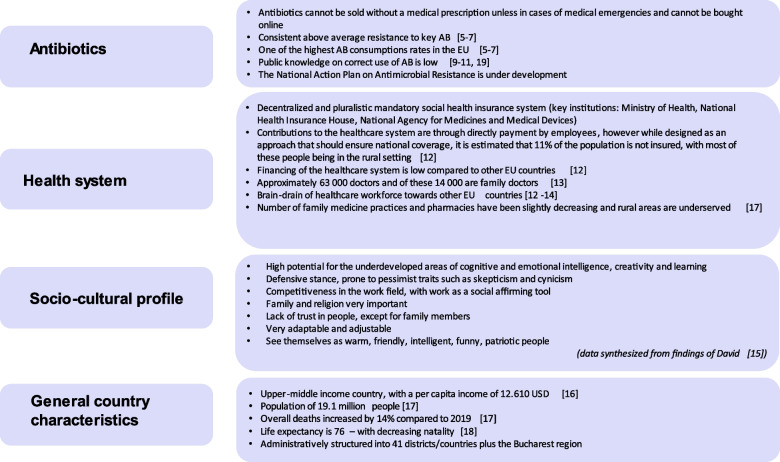
Key data on Romania

Figure 1 presents key details on the Romanian context [5–7, 9–19], references are also listed in the figure content).

This data suggests the need to curb antibiotics consumption including at the community level in Romania. This can be achieved by addressing related drivers, which result from antibiotics over-prescription by physicians, their dispensing without a prescription by pharmacists and their misuse by patients. These drivers are linked to knowledge, attitudes and practices of key stakeholders and would necessitate a greater understanding of factors that may predict, alter and promote responsible behaviours when it comes to antibiotic use [20]. In Romania there is very little research in this area. A previous study looked at the community pharmacists’ perceptions on their role in respect to AMR [21] and highlighted the perceived health system barriers that patients face in accessing care, the different negative incentives that determine pharmacist to push the limits of the law, the areas of improvement such as a more collaborative approach, the need to invest more in educational interventions for both pharmacists and the general public. These findings resonate with other results, which highlighted high prevalence of self-medication practices in the Romanian context [19]. While a study from 2019 conducted in one Romanian county -Mures, found that the majority of participants had good knowledge of antibiotic usage and the risks of self-medicating with antibiotics [22], recent Eurobarometer results seem to be less encouraging [9]. Among all EU countries, Romanian respondents had the lowest average number of correct answers with a decrease in knowledge compared to results from previous years [9]. Findings from the same survey show that doctors are considered as the main source to obtain information on antibiotics followed by pharmacists [9].

Previous systematic reviews [23–25] revealed several drivers that may influence GPs or family doctor’s decision to over-prescribe antibiotics. These can be structured as: i) physician-related factors such as complacency driven by a desire to maintain patient satisfaction and avoiding conflict, anxiety and fear, ii) patient-related factors which ranged from symptoms and co-morbidities, predisposition to complications that, based on the physician’s experience, demanded antibiotic treatment, pressure and a lack of patient education and awareness; and iii) healthcare system-related factors that placed pressure on time doctors could allocate to patient consultation, lack of policies and guidelines as well as lack of access to facilities (for either testing or treatment). In Romania, to our knowledge, there is no published research identifying the views of GPs and family doctors on the drivers that may influence their behaviour regarding antibiotic prescription or the antibiotic consumption and resistance problems in general.

Against this background we undertook a qualitative research study, with the aim to increase the understanding of how family doctors perceive the phenomenon of antibiotic consumption and antibiotic resistance in Romania, including how they see their roles in this respect. This research is particular timely, within the context of the development of the Romanian National Action Plan to tackle AMR. More widely presented results would also increase the knowledge base for countries that share similarities with Romania and are in the course of developing their national action plans.

## Methods

### Study design

This exploratory study used qualitative research methodology and content analysis to gain an in-depth understanding of the views and experiences of family doctors in Romania. The use of qualitative methodology was considered optimal as antibiotic misuse and over prescribing practices are complex phenomenon. Their drivers are routed in behavioural concepts, that merit a deeper understanding of the different stakeholders’ experiences and perceptions. Moreover, considering the limited research in the Romanian setting, qualitative methods allow to appropriately engage in these initial explorations.

### Data collection tools development and validation

The interview guide (see Annex 1) was developed by identifying key concepts through literature review as well as was guided by the overall research questions. Therefore, the questions aimed to gather contextual information such as opinions on the problem of unnecessary antibiotic consumption and antibiotic resistance in Romania and the role of family doctors in this area, drivers that would determine unnecessary requests and/or prescribing of antibiotics, potential interventions to curb antibiotic use and related considerations for their operationalisation and impact monitoring. The interview guide was developed in English and then translated in Romanian. It was tested on the first participant. The questions that were aimed to understand the resource needs and potential impact did not yield many reflections from the participant. This was also noticed in subsequent interviews and were deprioritised when interviewees expressed time-constraints for the interview.

### Recruitment

The recruitment strategy was guided by the aim to achieve maximum variation in terms of professional experience, geographical and gender distribution as well as variation in terms of urban/ rural place of work. This was envisaged to allow emergence of comprehensive themes. Different recruitment strategies were used to achieve this. A first strategy to reach relevant informants was to contact the National College of Physicians in Romania, who posted the invitation to participate in this research on their website. Secondly, purposive sampling was sought to achieve geographical and gender variation, by directly contacting doctors by phone through the publicly available contact data posted on the Romanian national insurance registry. Social media (Linked In platform) was also used to disseminate information on this research. In total approximately 200 family doctors were directly contacted over the phone to participate in this research between late August and end of October 2021. Of these 12 participated, the rest declining. All but one interview was performed by telephone (one was conducted face-to-face). The most common reason given for non-participation was the lack of time, some mentioning this is also due to heavier workload due to COVID-19, and in some cases a self-assessed lack of suitability, considering that an infectious disease specialist would be better suited to engage in an AMR research project. While doctors were contacted from all geographical regions, with similar efforts (telephoning at least five doctors in a certain county), no participation was attained from doctors from the East and South-East region of Romania. All potential participants were provided with study information ahead of giving informed consent to participate.

### Data collection

Interviews were conducted in September and October 2021, with one exception for an interview conducted in October 2020 (which was also used to test the interview guide). The interviews were conducted by the first author (IG) in Romanian language, and were audio recorded. The interviewer was native Romanian speaker. They lasted between 15 and 90 min, with an average of 30 min. Interviews were conducted within one- or two-days following confirmation of availability. Saturation was reached following 11^th^ interview, and one more interview was carried out to confirm this assessment. By saturation, we understand that interviews no longer involved provision of new information, and interviews were making smaller contributions compared to the previous ones. Participation was voluntary with no remuneration being offered.

### Data management and analysis

**Fig. 2:**
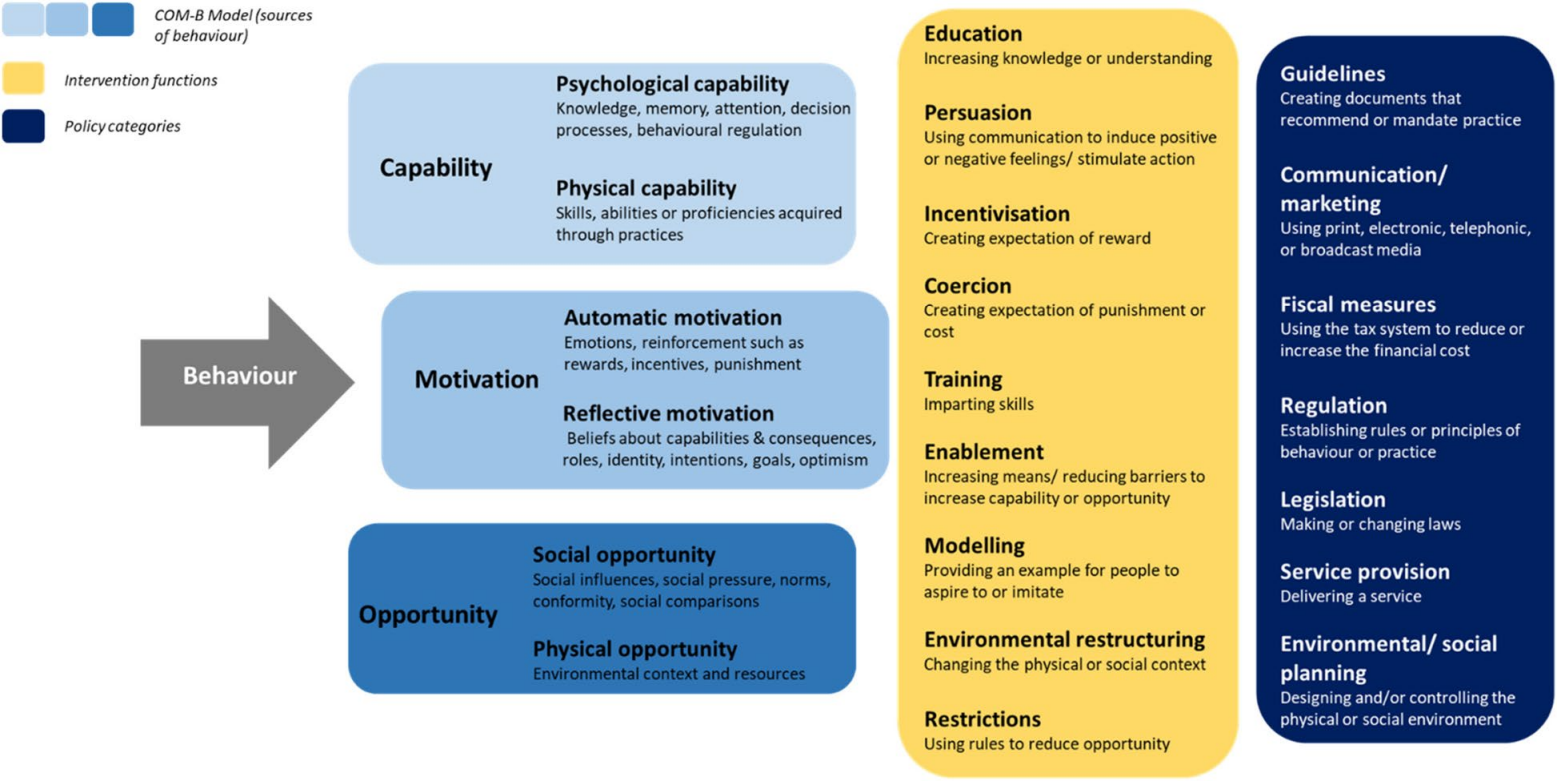
Adaptation of the Behaviour Change Wheel [27]. COM-B, Capability, Opportunity and Motivation Model of Behaviour

The audio records of the interviews were transcribed verbatim in Romanian. These were then translated into English. Content analysis was used to identify manifest categories and latent themes. Content analysis was used over thematic analysis, as there was an assumption that the data will allow description but may offer different levels of interpretation. Thematic analysis requires a more abstract level of abstraction and interpretation, which given the exploratory, reduced size of research may have raised issues around accuracy of interpretation of hidden meaning and potential loss of valuable information [26]. Excel was used to facilitate data analysis. Coding was employed to compile consistent and exclusive categories which reflected the manifest level. First, meaning units were identified and condensed. The condensed meaning units were grouped into sub-categories and lastly categories. Subsequently, these lead to sub-themes which were analysed to infer the underlying meaning. An attempt was made to articulate an overarching theme reflecting the latent meaning and overall interpretation of data. Findings were also mapped onto the domains of the Behaviour Change Wheel [27] which lists the domains of the Capability, Opportunity and Motivation Model of Behaviour (COM-B) as well as intervention functions and policy categories as shown in Fig. 2. This framework aims to foster an understanding of the behaviour that is meant to be modified, as well as offer a system to characterize a range of interventions, including policies, by linking them to specific behaviour components such as the capabilities, opportunities and motivations that form the behaviour. The COM-B has been used to aide evaluations of community-based interventions to tackle AMR as part of two systematic reviews [28, 29]. Therefore, we consider it opportune to align to these efforts and enable future comparable analysis to aid further research on the design, implementation and evaluation of behavioural change interventions. The analysis was led by one researcher. The analysis was discussed with the broader research team to interrogate findings and interpretation.

### Ethical considerations

The research was reviewed and approved by the National Bioethics Committee of Medicine and Medical Devices in Romania – approval registration no 3SNI on 17.02.2020. Participants were informed about the nature of the research, their right to refuse or stop participation at any moment. All participants received and signed the corresponding informed consent form.

## Results

In total the research findings draw on responses of 12 participants with ages between 35 and 71, of which 42% were female. Only one participant worked in the rural setting. Family doctors worked in direct contractual agreement with the National Insurance House in Romania and did not work through private clinics.

Two main subthemes emerged following data analysis. These were: i) factors affecting antibiotic consumption and prescribing and ii) potential interventions to tackle antibiotic consumption and antibiotic resistance. An overarching theme was also articulated: family doctors in Romania see their role differently amongst themselves when it comes to antibiotic resistance and see lack of patient education or awareness as one of the major drivers of antibiotic consumption.

The results of the content analysis are presented in Table 1. We then map identified factors and interventions to tackle antibiotic consumption and resistance as expressed by family doctors in Romania on the Behaviour Change Wheel [27]. Considerations are explored in the discussion section.

**Table 1:** Overview of manifest and latent meaning findings

**Manifest meaning (categories in bold and subcategories plain text)**		Latent meaning
	Subthemes	Overarching theme
**Family doctors related factors** • Different understanding of the magnitude and causes of antibiotic consumption, resistance and prescribing • Interpretability of existing guidelines, lack of diagnostics, reliance on experience and complacency	Factors affecting antibiotic consumption and prescribing	Family doctors in Romania see their role differently when it comes to antibiotic resistance and see lack of patient education or awareness as one of the major drivers of antibiotic consumption
**Patient related factors** • Undue pressure and transfer to another doctor • Poor medical knowledge • Opportunities to acquire antibiotics from other sources • Lack of treatment adherence		
**System and contextual factors**		
**COVID-19 pandemic exacerbated antibiotic consumption and hinders other public health campaigns**		
**Local communities provide better opportunities for greater engagement with the general public**		
**Enhancing medical knowledge for healthcare professionals**	Potential interventions to tackle antibiotic consumption and antibiotic resistance	
**Statistics and feedback loops to be used with caution**		
**Public education**		
**Controls in pharmacies**		
**Antibiotics packaging**		

### Factors affecting AB consumption and prescribing

**•** ***Family doctors related factors***

### Different understanding of the magnitude and causes of antibiotic consumption, resistance and prescribing

Participants offered different perceptions on the extent of the antibiotic consumption and AMR in Romania. While some were aware of the current official statistics, some mentioned the situation improved drastically as the result of changing of legislation, which limited pharmacy sales without prescription, and this is no longer an issue. Another participant mentioned that one cannot notice necessarily the extent of the problem from direct medical practice.

*INT2 (female, 35 years old): We are in second place in Europe when it comes to antibiotic resistance. This is a very worrying situation*.

*INT4 (male, 50 years old): I don’t think this [AMR] is a problem. It used to be a problem in the 90 s when antibiotics were dispensed without prescription*.

There were also varying opinions about the family doctors’ role and contribution to the phenomenon with some considering there is over prescribing from the family doctors and paediatricians, that the family doctors have a small influence or that other specialities would be better placed to engage when it comes to AMR. Furthermore, the problem was perceived by some participants as being more prevalent and serious due to the hospital setting where drug-resistant bacteria emerge, and is less of a community driven phenomenon.

*INT2 (female, 35 years old): There is an abuse of prescribing antibiotics, it’s enormous .. and the paediatricians.. I think in first place are the family doctors and the paediatricians. and the rest of specialities follow.*

*INT5 (male, 53 years old): Oh dear, but you are asking a family doctor, you should be asking a specialist, me as a family doctor what can I tell you.. I don’t know what to tell you.. I am not working with antibiograms .. more like this, from my medical knowledge. This is a topic for an infectiousness.*

### Interpretability of existing guidelines, lack of diagnostics, reliance on experience and complacency

Existing guidelines were considered useful. It was stressed that their role is to guide the doctor, which ultimately needs to decide whether it is appropriate to follow provisions. The fact that guidelines are open to interpretation, meaning that the provisions are broad and not mandatory, brings strengths and weaknesses. It was mentioned that it may be beneficial to have more direction on which antibiotics should be used first. Furthermore, better diagnostics capacities would also help doctors.

*INT12 (male, 61 years old): The guideline remains a matrix, which you can use or not, that’s why it’s a guide. But the treatment must be individualised, and here is where the experience of that doctor is important.*

One participant also mentioned a situation where family doctors may give out antibiotics, not because of lack of knowledge but rather out of complacency.

INT9 (male, 47 years old): There are situations where family doctors give out antibiotics not because they don’t know but because of complacency.

**•** ***Patient related factors***

### Undue pressure and transfer to another doctor

Family doctors perceived that patients could exert pressure by asking for antibiotics and eluding to moving to another doctor in case of dissatisfaction. However, by building rapport and by strengthening patient-doctor communication, it was considered that patients would ultimately understand they would be prescribed antibiotics only when needed.

*INT6 (male, 56 years old): The influence of family doctors is very small, because me for example as a family doctor I am against abusive use of antibiotics, but the patient will just go to another doctor.*

### Poor medical knowledge

Patients’ knowledge on use of antibiotics and risks associated with misuse was considered to be insufficient and considered a major source of antibiotic consumption. This may also have been perpetuated by past obsolete medical practices. Participants were also asked whether the social-economic situation of a patient may influence their decision to take antibiotics, but this was not seen as a major factor compared to the educational component.

*INT2 (female, 35 years old): A lot of medical education, two folds – once with the doctors, a re-evaluation of the indications and ways of prescribing, and secondly patients’ education, because many times, these antibiotics are prescribed at the request of the patient. There is a certain type of pressure for which you need to be very prepared for. Patients in Romania, the big mass of people, they live with the conviction that the antibiotic is antipyretic, analgetic .. when you tell them there is no reason why you should be taking antibiotics when you have a cold, the answer is ‘Yes, but I have a fever’ or ‘I have a headache’*

### Opportunities to acquire antibiotics from other sources

The lack of knowledge and assumption that antibiotics should be taken for any cold-like symptoms, is also credited to be the cause of patients starting the treatment at their own volition by obtaining antibiotics either from pharmacies or from the emergency setting.

*INT7 (male, 71 years old): [...] they say for them it only passes with antibiotics and they insist, I am not a proponent of antibiotics but […] still in our country, in a pharmacy, if the person insists, they are dispensed an antibiotic without a medical prescription even if now it’s more regulated, they still can get it, if not, they borrow from their neighbours ..*

Furthermore, patients are also thought to take antibiotics from acquaintances that may not have any medical training. Patients would then need to return the ‘borrowed’ antibiotics.

*INT3 (female, 60 years old): The law should be respected – they come though having already started the treatment: ‘I already started taking Augmentin, could you please give me some to continue and I also want to return some to my neighbour’ […] There are situations where people want to have an antibiotic at home, or to have a spare if they go abroad.*

### Lack of treatment adherence

Obtaining antibiotics, even if in small quantities, from other sources leads patients to arrive at the family doctor once they finish these ‘emergency’ doses. In such situations the doctor reported feeling forced to prescribe antibiotics so that they can have a minimum number of days to continue treatment. While less frequent, lack of adherence to following through the treatment was also mentioned, with some patients arbitrarily deciding to stop taking antibiotics as soon as symptoms improved and save the pills for other occasions.

*INT7 (male, 71 years old): Yes, they come and they say they took amoxicillin .. and then so that you are not in the situation to create a sensitization to that antibiotic, you need to continue at lease 2-3 days, if they took it, at least to have the full treatment;*

**•** ***System and contextual factors***

Participants reflected that broader system and contextual factors were relevant, for example the pharmacies were perceived to be dispensing antibiotics without prescriptions in high numbers therefore contributing to misuse of antibiotics.

*INT1 (female, 62 years old):.. I noticed that in the big pharmacy chains they no longer give out antibiotics, but in the small ones they still do, and they no longer come to the family doctor and they say that the ‘pharmacy doctor’ gave them the antibiotics.*

Another factor that may impact decision to prescribe antibiotics was the patients’ living situation, suggesting that a patient located in a rural setting may have difficulties in accessing medical care and return to ‘control visits’, therefore it may be more prudent to prescribe antibiotics.

*INT10 (male, 53 years old): it’s one thing to treat someone in town, 300m from the practice and it’s something different to treat someone who lives 20 km in the woods somewhere. That’s why I’m saying that treatment individualisation needs to be very well thought through.*

### COVID-19 pandemic exacerbated antibiotic consumption and hinders other public health campaigns

The COVID-19 pandemic is thought to have exacerbated antibiotic prescribing and consumption due to the early recommendation of prescribing azithromycin for COVID-19 patients. Most of participants expressed a lack of understanding why this was recommended by the Romanian authorities. Furthermore, at the time of the interviews, participants also considered that due to the unfolding pandemic, no other public health- oriented campaign (such as one to educate the public) would be feasible or efficient due to the ongoing vaccination campaigns.

*INT2 (female, 35 years old): There needs to be an educational campaign, but now it’s not possible as another campaign should be taking place…I never understood though why we were asked to prescribe azithromycin for COVID-19.. honestly, nobody explained.*

It was also expressed that while hygiene efforts intensified and this may have a positive effect to reduce bacterial infections, not much can be learned from the current COVID-19 behaviour change interventions, as these were not seen to be effective (e.g. encouraging vaccination).

INT5 (male, 53 years old): I think it’s an inefficient campaign [about the COVID-19 vaccine], without much planning.. mostly it’s disinformation.

### Local communities provide better opportunities for greater engagement

It was stressed that local communities provide better opportunities to engage and tailor approaches for better uptake.

*INT12 (male, 61 years old): I don’t think this is necessarily a system thing more about local actions […] local initiatives are very good because they account for specificities and someone that actually works locally, is invested personally and is known, they should be involved to see this through.*

### Potential interventions to tackle antibiotic consumption and AMR

#### Enhancing medical knowledge for healthcare professionals

Activities such as round tables and symposiums have been mentioned as interventions that would aide with improving or refreshing medical knowledge on antibiotics. These events should occur periodically, have a multidisciplinary composition, although most of the time only doctors of different specialities were mentioned as potential participants. Preferably these events should not take place in an online format (which was found to have low participation and engagement).

*INT3 (female, 60 years old): More round-tables discussions should be organized. And periodically, not only once. Infectious disease specialists, Ministry of Health representatives, pharmacists and dentists should be invited.*

### Statistics and feedback loops to be used with caution

While it may be useful to have access to statistics which could be supplied through periodic newsletters, when it comes to prescription data it is important not to judge numbers disjointly from the medical cases history as antibiotic prescriptions could have been necessary. Participants expressed worries that an approach to limit antibiotic prescribing, as employed in other western European countries may not be suitable, as they have had patients returning to Romania with very serious infections for which they received paracetamol.

INT12 (male, 61 years old): A simple monthly doctors’ prescriptions report won’t say whether the antibiotics were necessary or not, it won’t express seriousness.

*INT10 (male, 53 years old): We know there are countries like Sweden, the Nordic countries, England which are more severe, but also from there, I had patients coming with sinusitis that were poorly managed, with a perforated timpan because of the reticence to keep things under control with an antibiotic – this isn’t a good approach either; the approach should be to individualise the treatment.*

### Public education

Educating the public has overwhelmingly been suggested by all participants as a potential intervention to tackle AMC and AMR. Suggested channels to conduct educational campaigns vary from family doctors’ practice, pharmacies, schools, social media and most importantly through TV. However, participants stressed that impact would not be noticeable in the short timeframe.

*INT10 (male, 53 years old): Doctors need to be trained through materials, be notified about new medications, a lot of population education ..but this is a continuous effort, that will take years and maybe then a generation will appear which will take less antibiotics.*

Children have been mentioned as a good target group, especially secondary and high-school students so that good behaviours could be inculcated early. However, challenges would consist of the reduction in numbers or the lack of school doctors. Games and cartoons could be used to convey medical messages in a friendlier format.

*INT2 (female, 35 years old): It would be wonderful, that this effort starts with young ages. Children of course, don’t have the power of deciding, but it does prepare them for the future. Secondary school would be best.*

However, as efforts to educate the public and healthcare professionals increase, there needs to be special attention to the growing trend of distrust in science.

INT12 (male, 61 years old): I think there is a challenge with the entire perception on science… Trust in science, less dogma.

### Controls in pharmacies

Greater controls in community pharmacies was suggested due to suspicions that antibiotics are dispensed without prescription in cases that may not be emergencies (which is the current law).

*INT11 (male, 59 years old): there is a complicity from the part of pharmacies […] it has changed only partially because we still are living with this tendency from the pharmacist to compete with the doctor’s profession, this is my opinion.*

### Antibiotics packaging

One participant drew a parallel with tobacco and alcohol labels and mentioned that learning from this field could be applied to warn patients on the dangers of antibiotics misuse.

*INT7 (male, 71 years old): well for children we could do some cartoons related to antibiotics, and similarly for adults, just like we do for cigarettes packaging, for alcohol, circulation accidents.*

## Discussion

This was the first study aimed to offer a greater understanding of the family doctor’s views on the factors that may affect antibiotic consumption and emergence of resistance as well as explore potential interventions that could be implemented in the Romanian setting. Our findings suggest that the perceived factors span across the capability, opportunity and motivational domains of the behaviour change wheel and could be addressed through a variety of interventions. A synthesis of the factors and of the related, identified and potential additional interventions are presented in Table 2. The identified driving factors are overall consistent with findings from other settings as presented in several systematic reviews [23–25].

**Table 2:** Proposed intervention functions and policy categories for the identified factors

COM-B Model Domains	Identified factors	Intervention functions	Policy categories and interventions identified by participants	Detailed interventions including examples of additional potential interventions or policies from other settings
**Physical capability**	Lack of diagnostics	Incentivisation		• Enhanced access to antibiograms • Availability of rapid diagnostic tests • Enhancing physical capability for parents to perform rhinopharyngeal clearance
**Psychological capability**	Different understanding of ABC and AMR magnitude	Enablement	• Periodic updates with statistics	• Enhanced surveillance networks • Auditing and feedback mechanisms on GP prescriptions • Newsletters with: • statistics • case studies of best practices
	Interpretability of guidelines, reliance on experience and commodity	Education Training	• Guidelines that are more precise	• Job aids, prescription pads and infographics • Updated guidelines
**Reflective motivation**	Perceptions on easiness for patients to start treatment elsewhere (through pharmacies or emergency care)	Education Persuasion Coercion Restriction	• Education campaign for the public • Greater controls in pharmacies	• Education campaigns: i) For the public: ○ TV, radio and social media clips/spots ○ Posters, leaflets, brochures, billboards, bus tails, bus stop posters, interior bus signs ○ Roadshows ○ Theatre plays ○ Interactive website with videos and other information materials, pledging system and competitions (e.g. for creating videos to disseminate educational messages) ○ Mobile Apps ii) For health care providers: ○ Round tables and symposiums ○ Pledge campaigns (e.g. Antibiotic guardian) ○ Training courses iii) For children and youth: ○ Games (such as ebug) ○ Special school classes delivered by healthcare professionals or peers (older students) ○ Wet and dry lab activities ○ Cartoon on TV ○ Science-show iv) For patients: ○ GPs and pharmacist education sessions ○ Call-back by phone to check adherence to treatment ○ SMS based reminders ○ Antibiotic packaging with warning messages • Greater control mechanisms in pharmacies and communication of findings (to check accuracy of different perceptions)
	Patients’ lack of knowledge on risks and lack of adherence to treatment	Education Training Persuasion	• Education campaign for the public	
	Family Doctors have an important role as educators	Education Training Modelling	• Education campaign for the public	
**Automatic motivation**	Maintaining good relations with patients and fear of losing them	Education Persuasion	• Education campaign for the public	
**Physical opportunity**	Lack of access or continuity of care in rural settings	Environmental restructuring	• Treatment individualisation	• Mobile clinic or pharmacy services • Making use of eHealth apps to ensure follow-up
	COVID pandemic brought higher ABC	Education Coercion Restriction	• COVID-19 treatment guidelines • Education campaign for the public	• Clarity on COVID-19 treatment recommendations • Increased surveillance for emergence of resistance to azithromycin
	AMR occurs mostly due to hospital acquired infections	Environmental restructuring		• Antibiotic stewardship interventions for hospital setting
	Patients may see antibiotics as preventive treatment and obtain medical advice from their social network rather than medical professionals	Education Persuasion Coercion Restriction	• Education campaign for the public	
	Communities enable action to tackle ABC	Environmental restructuring	• Tailoring and piloting campaigns locally	

Regarding needed capabilities, family doctors’ in Romania identified difficulties linked to lack of diagnostics capacities. This is consistent with other research that called for a need to have rapid tests that would enable diagnostics at point of care and would complement empirical prescribing practices [30]. Factors that refer to psychological capabilities relate to the different perceptions of antibiotic consumption and AMR, the interpretability of guidelines and the need to rely on experience as well as potential ‘commodity’ of doctors to prescribe antibiotics. Participants suggested interventions such as periodic updates with statistics -judged jointly with diagnosis, and updating of guidelines. Other interventions that have been implemented in different settings and that could enhance the relevant phycological capabilities are the supplying of job aids, prescription pads and infographics for a quicker identification of relevant information [28, 31]. Surprisingly, the interviews did not reveal particular concerns with the time doctors can dedicate to their patients’ consultations. Considering the Romanian context and the doctors’ exode to other European countries, this could have emerged as a barrier to providing patient counselling and education. In Romania, there are approximately 63 000 doctors and of these 14 000 are family doctors [13]. Since joining the EU in 2007, there has been a significant brain drain, with many doctors emigrating [14]. This is estimated to bring increasing constraints on providing primary health care. However, the lack of emergence of this finding should be interpreted with caution, as the interviews were time-constrained—this in itself showcasing doctors’ limited availability.

Factors affecting reflective and automatic motivation relate to: i) perceptions on easiness for patients to start treatment elsewhere, ii) their lack of knowledge on risks and lack of adherence to treatment, and iii) the perceived pressure that they may move to another doctor should they feel their needs for antibiotics are not served. One positive factor is that family doctors do see themselves as having an important role as educators. However, some participants didn’t feel that family doctors had a central role, this being more the realm of infectious disease specialists, or doctors that work in the hospital where resistance to antibiotics is more evident. Recent systematic reviews list a series of interventions that could be implemented to conduct educational campaigns in a community setting [28, 29]. These could consist of public facing campaigns (through a series of traditional media channels as well as more interactive ways such as roadshows and theatre plays), competitions, educational campaigns targeting health care providers, including round tables and symposiums, pledge campaigns such as the UK Antibiotic Guardian campaign or more traditional training courses. Promising interventions meant to reach children and youth could consist of games, special school classes delivered by healthcare professionals or peers (older students), wet and dry lab activities, TV cartoons or school-based science-shows [28]. An example of successful game intervention is the ebug –a programme originating from the UK that has been widely introduced across the EU and has been comprehensively evaluated [32]. Patients that come to ambulatory care could be approached through GPs and pharmacist education sessions, interventions consisting of call-back by phone to check adherence to treatment or short messages based reminders. However, the findings from these systematic reviews showed varying degrees of effectiveness with multifaceted interventions that combine education, restriction and training having a greater impact [28]. As suggested by one participant, antibiotic packaging with warning messages could be explored to flag the risks linked with misuse of antibiotics. Participants also mentioned the need for greater control mechanisms in pharmacies. Previous research in Romania which captured the pharmacists’ perceptions on antibiotic resistance, does support observations that there are cases when pharmacists would make use of the law to dispense antibiotics without a medical prescription [21]. However, the extent and frequency of these practices are not clear. Comparing the perceptions expressed by the pharmacists and the doctors separately, there is a lack of trust in the practices of prescribing and dispensing antibiotics on both sides. Interventions aimed to increase trust could be explored through organising inter-professional collaborations (round-tables, trainings and seminars) and disseminating periodic data on antibiotic prescriptions and sales through pharmacies.

When it comes to the opportunity domain, participants stressed mostly physical opportunity related factors such as lack of access or continuity of care in rural settings, the negative impact of the COVID-19 pandemic, the gravity of hospital acquired infections as well as the enabling power of communities. Interventions to address these factors would require system-based approaches such as ensuring infrastructure to cover the gaps in continuity of care. Mobile clinics or pharmacy services could be explored as well as deploying eHealth solutions that could facilitate monitoring and follow-up. The reported early COVID-19 treatment recommendations, contained a somewhat lack of clarity on the use azithromycin, leading to it being prescribed in early 2020 which may result in resistance to this antibiotic and potentially infer cross-resistance to other antibiotics. This emphasizes the need for increased surveillance to quickly identify any emergent trends. While the guidelines issued in November 2020 clearly discouraged the use of azithromycin [32], the latest ECDC report shows that Romania ranks first when it comes to total consumption of antibiotics for systemic use within the EU. This data shows a set-back to the levels the country experienced ten years ago [7]. It is difficult to speculate on the exact causes of this rise; however, it is expected that the COVID-19 pandemic played a major role. Potential explanations could be: a slow dissemination of the updated clinical guidance, perceived secondary bacterial infections in patients with COVID-19 coupled with difficulties in accessing healthcare facilities and laboratories that might have shifted capacities to respond to the pandemic, potential perceived economic hardship experienced by patients which in turn might have deterred doctors and pharmacists to be less strict with prescribing or dispensing of antibiotics in an effort to reduce their hospital-related expenses. These potential pressures could also explain the high consumption of broad-spectrum antibiotics in Romania. Previous research has shown that increasing pressure on family doctors leads to increase in broad-spectrum antibiotics prescriptions [33]. This is a worrying trend as broad-spectrum antibiotics drive AMR more compared to the narrow-spectrum ones [34]. However, with limited practice and capacity to perform antibiograms, it may be difficult for a family doctor to prescribe a narrow-spectrum antibiotic that may not match the causative pathogen, considering the medical risk-assessment as well as cultural considerations related to the doctor-patient relationship. Tackling AMR in hospital settings will require a full antibiotic stewardship package. Data and past cases reported in the Romanian setting do support the perceptions expressed by the family doctors regarding the need to act against AMR in a hospital setting. In addition to the above mentioned ECDC reported data, high profile cases were reported in the press in recent years having to do with substandard quality of disinfectants used in hospitals [35]. Lastly, communities have been identified as enablers to interventions against AMR. Their early involvement is needed in the design, tailoring and piloting of campaigns. However, to ensure this, levers for enabling a participatory community process needs to be further identified with a starting point of agreement on the problem, identification of community needs and ‘assets’ as they relate to the problem, and consensus on the underlying measures that should be explored.

The overarching theme signalled that there is a perceived lack of patient education leading to unnecessary antibiotic consumption. As patient education is also facilitated by healthcare professionals’ interactions, this leads to a potential need for strengthening the doctor-patient relationship. Previous research in Romania flagged that patients may not take for granted a doctor’s legitimacy and they tend to seek confirmation regarding the doctor’s expertise based on treatment success and feedback from other patients [36]. Patients also increasingly seek additional information online and introduce alternative treatment methods or not follow treatment at all, in an attempt to gain further control on their disease [36]. As eluded by one of the participants, there is an increasing distrust in science that was amplified by the pandemic response which was challenged by disinformation and enhanced by the increase in social media use. These developments and experiences may transform the norms around the doctor- patient relationship, requiring a higher degree of engagement with the patient to meet the increasing patients’ agency. As access to greater amount of information is enabled by technologies, it may become necessary for medical professionals to ‘train’ patients on how to assess such medical data while ensuring that patients’ also understand that greater agency may imply greater responsibility and accountability from their part.

All these considerations need to be underlined by continuous efforts to bridge the ‘intention-behaviour gap’ [37]—recognising the need to adopt a certain behaviour even if accompanied by a degree of motivation, may not suffice to effect behaviour change if certain barriers are in place. This, corroborated with findings from recent systematic reviews, would suggest the need for deploy a mix of interventions (rather than a short selection) so that factors across the behaviour-change-wheel can be targeted comprehensively.

The challenges highlighted above in regards to the doctor-pharmacist and doctor-patient relationship may also be explored through cultural lenses. Using Hofstede's model of cultural dimensions,^1^ Deschepper et al. [38] found indications that countries with high Power Distance (high level of hierarchy) – and high Uncertainty Avoidance (low tolerance for uncertainty and ambiguity)- such as Romania, seem to experience greater antibiotic use. Within the context of the patient-doctor relationship, as well as relations between professionals, the high Power-Distance would translate in deference towards the doctor, and a lesser need for the patients to be involved in medical decision-making [38]. However, our findings would suggest a more complex phenomenon, as while decision-making may be one-sided, there are indications of patient pressure in relation to medical decisions. This together with the emergent distrust in science, may signal a shift of patients type from deferent or ignored towards critical, rather than towards involved.^2^ Therefore, it is important to empower doctors to steward shifts in patients’ types, by equipping them with correct information and trusted platforms they can recommend to patients that would like to become more health literate. A high Power-Distance and Uncertainty Avoidance, is also thought to impede doctors from acknowledging situations of uncertainty (e.g. admitting not knowing whether an infection is caused by a virus or a bacteria) out of fear of patient losing confidence in their medical expertise [38]. This dovetails lightly with findings from our study, with doctors being concerned of losing the patients that may choose to visit another doctor that would prescribe antibiotics more willingly. However, this may be more driven by economic considerations rather than fear of being considered less competent. In summary our findings suggest that cultural dimensions may play a role in antibiotic use in Romania, however these should be further explored in studies that would benefit from a larger sample size.

### Methodological considerations

Trustworthiness of this research was sought by enlisting measures to ensure: credibility, transferability, dependability, and confirmability. Credibility was fostered by having interviews performed by an experienced interviewer, a Romanian native speaker familiar with contextual specificities such as social and health system, cultural, economic, policies and political environment. The interviewer did not know any of the participants, sought to create rapport and provided information about herself and the nature of the research. Limitations of the study are linked to the geographical, work sector and age representation of participants as well as constraints with the fact that the research was conducted during the hight of the COVID-19 pandemic. While the interviewer was based in Romania, the majority of the interviews, due to the ongoing pandemic, had to be performed over the phone. This posed challenges in terms of reach as well as inability to notice non-verbal communications or ques. Furthermore, in some cases participants mentioned from the start that they cannot devote more than a certain (oftentimes limited) time for the interview. All participants worked as part of their own practice (not part of a private clinic), only one participant was working in rural setting and overall the participants were experienced physicians, therefore the voices of young doctors just entering the field of practice, may not necessarily be reflected in these findings. Transferability was fostered by providing detailed descriptions of the context which would allow other researchers to assess whether findings could be applicable to other settings. Efforts were made to ensure clear methodological descriptions as well as maintenance of study records to allow traceability and repeatability of the study. These were meant to ensure dependability. Confirmability was sought though efforts to foster data neutrality. The interviewer exhibited deliberate naiveté when conducting the interviews and asked probing questions to ensure accurate understanding. Potential sources of bias could arise from the interviewer’s previous research which aimed to capture views on AMR in Romania from the perspective of pharmacists. However, the interview guide was designed to have open-ended questions which would avoid as much as possible the steering of answers.

## Conclusion

The study revealed that family doctors in Romania have varying perceptions on AMR as a national issue. Identified factors contributing to ABC and AMR were related to the perceived behaviour of family doctors or patients as well as to the health system, local contexts and the COVID-19 pandemic. Several potential interventions to tackle these determinant factors emerged, however they seemed to revolve mostly on efforts to educate patients or the public. Participants did not offer conclusive feedback on operationalisation of interventions. This exploratory research provides key perspectives and direction to facilitate further research on potential interventions to be introduced, to successfully address antibiotic resistance in Romanian-or similar settings.

## Supplementary Information

**Additional file 1.Annex 1.** Interview Guide.

## Data Availability

The data from interviews used and/or analysed during the current study are available from the corresponding author on reasonable request – respecting the confidentiality and data protection requirements.

## Abbreviations

AMR: Antimicrobial resistance
ABC: Antibiotic consumption
ECDC: European centre for disease prevention and control

## Footnotes

1 The cultural dimensions in the model are: Power Distance, Individualism, Masculinity, Uncertainty Avoidance and Long-Term Orientation.

2 Pechère et al. [39] proposed four patients’ typologies: i) involved patient—involved in their own medical care, deciding jointly on best approaches; ii) deferent patient—deferent to their doctor and not making joint decisions; iii) ignored patient – reduced interaction with the doctor, feeling ignored but less likely to disagree with the doctor or consider the doctor incompetent; iv) critical patient—doubt the doctor's competence and expertise.
