# Supplementary material for: Family doctors’ roles and perceptions on antibiotic consumption and antibiotic resistance in Romania: a qualitative study
Source: BMC Prim Care. 2023 Apr 10;24:93. doi: 10.1186/s12875-023-02047-z (PMC10084585; doi:10.1186/s12875-023-02047-z)
Supplement: Supplementary file 1 — Additional file 1.Annex 1. Interview Guide. [file 12875_2023_2047_MOESM1_ESM.docx]

**Interview Guide**

**Background information from the key stakeholder:**

- Age:
- Gender:
- Place of current employment:

**Possible interview questions:**

**External context conditions**

- What do you think about antibiotic resistance in general? What are your thoughts about the situation in Romania?
- When it comes to key stakeholders in relation to ABR and ABC (AB Consumption), who comes to mind? How do they relate to these issues? What do you see them doing in this respect? How do you think they can be involved/influenced to take positive action?
- What is your role in relation to antibiotics? Have you noticed any changes when it comes to your profession, the way other stakeholders perceive your role in general?
- How do you see the role of the national structures like the Ministry of Health or other institutions/ professional associations/ public/patient associations in respect to antibiotic consumption and antibiotic resistance?
- What do you think should be the relationship between these bodies and family doctors in relation to antibiotics and antibiotic resistance?
- Can you give an example of some pieces of legislation that you know of and are relevant for ABR control?
- What do you think should be done in terms of legislation for better ABR control? [How would this be done? Barriers/Enablers]

**Assumptions regarding the ABR problem and implementation of interventions**

- In your experience is it common that patients ask to prescribe AB?
- What type of antibiotics do they usually ask for? (Points to consider: pathologies, injection formulations, parents asking without prescription?)
- How would you say it is the knowledge of the patients on AB – do they know what they are for, how to take them and for how long?
- How do you think they see the family doctors when it comes to antibiotics?
- (In your practice) is it common to perform an antibiogram for patients that come to the family doctors?)
- Could you tell me of an instance when you had a collaboration with a colleague (pharmacist or another doctor) on the subject of antibiotics?
- What do you think are some of the social and economic factors that may determine someone to ask for AB without prescription/ determine a healthcare professional to prescribe/dispense AB without prescription in a situation when it is not very clear there is a medical need for it?
- In terms of getting information on antibiotic resistance what is your current practice? (Points to consider: Guidelines? Protocols? How do you find them – up to date? Statistics in Romania)

**Impact**

- What you think a good intervention address the problem of ABR and ABC should achieve in the long-term?
- How could we measure that?
- How feasible do you think these interventions could be considering the Romanian context?
- What would you say at the moment are the biggest challenges for a family doctors to fulfill all their potential in respect to developing and implementing such interventions?
- How do you think this issue should be addressed? What would you like to see improved considering all that we have discussed in respect to antibiotics?

**Resources**

- What are some of the resources that may be needed to implement interventions to achieve the changes we just discussed?
- Who would be in a position to supply such resources?
- Who are the stakeholders who should be involved in the development, implementation and evaluation of such interventions? Who do you think should be informed about this? Who do you think may not agree with this intervention and may try to oppose it?

**Concluding questions**

- What should be done to better support the family doctors’ role in respect to proper antibiotics management?
- Is there anything else you would like to add?
